# *In silico *identification of opossum cytokine genes suggests the complexity of the marsupial immune system rivals that of eutherian mammals

**DOI:** 10.1186/1745-7580-2-4

**Published:** 2006-11-10

**Authors:** Emily SW Wong, Lauren J Young, Anthony T Papenfuss, Katherine Belov

**Affiliations:** 1 Faculty of Veterinary Science, University of Sydney, Sydney, New South Wales, Australia; 2 School of Chemical and Biomedical Sciences, Central Queensland University, Rockhampton, Queensland, Australia; 3 Division of Bioinformatics, The Walter and Eliza Hall Institute of Medical Research, Melbourne, Victoria, Australia

## Abstract

**Background:**

Cytokines are small proteins that regulate immunity in vertebrate species. Marsupial and eutherian mammals last shared a common ancestor more than 180 million years ago, so it is not surprising that attempts to isolate many key marsupial cytokines using traditional laboratory techniques have been unsuccessful. This paucity of molecular data has led some authors to suggest that the marsupial immune system is 'primitive' and not on par with the sophisticated immune system of eutherian (placental) mammals.

**Results:**

The sequencing of the first marsupial genome has allowed us to identify highly divergent immune genes. We used gene prediction methods that incorporate the identification of gene location using BLAST, SYNTENY + BLAST and HMMER to identify 23 key marsupial immune genes, including *IFN-γ*, *IL-2*, *IL-4*, *IL-6*, *IL-12 *and *IL-13*, in the genome of the grey short-tailed opossum (*Monodelphis domestica*). Many of these genes were not predicted in the publicly available automated annotations.

**Conclusion:**

The power of this approach was demonstrated by the identification of orthologous cytokines between marsupials and eutherians that share only 30% identity at the amino acid level. Furthermore, the presence of key immunological genes suggests that marsupials do indeed possess a sophisticated immune system, whose function may parallel that of eutherian mammals.

## Background

The marsupial and eutherian (placental) lineages diverged approximately 180 million years ago. Marsupials are chiefly distinguished from other mammals by their unique reproductive strategies, with young born in an immature state with only the most rudimentary neurological and immunological systems [1]. At birth, the animal manoeuvres its way to a waiting teat, where it attaches until it reaches a state of maturity that allows it to function independently. Marsupials possess lymphoid tissue and cellular components that are structurally similar to those of other mammals. Key antigen receptor and recognition molecules including Major Histocompatibility (MHC) Class I, II and III [2], T Cell Receptors alpha, beta, gamma and delta [3,4], Toll-like receptors [5] and immunoglobulins [6] have been characterized.

However, conventional experimental strategies using degenerate primers for reverse-transcriptase polymerase chain reaction (RT-PCR) and heterologous probes for screening genetic libraries have only identified the most phylogenetically conserved immune molecules, with cytokines proving particularly difficult to isolate [7]. To date, only eleven cytokines including one receptor have been cloned from marsupials. They include tumour necrosis factor alpha (TNF-α) [8,9], lymphotoxin (LT) -α and -β [10,11], Interleukin IL-1β [12], IL-1R2 [7], IL-5 [13], IL-10 [14], leukemia inhibitory factor LIF; a member of the IL-6 family [15] and three type I Interferon (IFN) genes [16]. These cytokines show relatively high levels of identity compared to their eutherian homologues. Previous attempts to isolate the more divergent T-cell derived cytokines that orchestrate adaptive immunity such as IL-2, IL-4 and interferon-γ have failed [7,17].

Identification of divergent marsupial immune genes is important for two reasons. Firstly, unsuccessful attempts to isolate T cell derived cytokines in the laboratory has led some authors to suggest that the marsupial immune system is 'primitive' and does not possess the level of complexity demonstrated by eutherians such as humans and mice. The fact that some T cell driven responses are also aberrant adds to this argument. Marsupials appear to have delayed skin graft rejection [18] and antibody class switching [19], together with an apparent lack of an *in vitro *Mixed Lymphocyte Response [20]. Elucidation of genes involved in specific immunity will help us to determine whether the apparently 'simple' immune responses generated by marsupials are genetically hardwired.

The second reason for identifying divergent immune genes in the marsupial genome is to develop marsupial specific immunological reagents. To date, most assay systems employed to characterise cells and their function rely on eutherian reagents or culture techniques developed in eutherian species. Where low levels of cross reactivity exist between marsupials and these model species, the usefulness of the data generated from such assays is limited. Identification of key cell markers, such as CD4 and CD8 will allow us to generate marsupial-specific reagents, which would then be used to gain a better understanding of the marsupial immune response.

Difficulties associated with identifying rapidly evolving cytokines are not limited to marsupials. The chicken *IL-2 *gene took seven years of focused effort to identify [21], and was eventually found using expression strategies and not heterologous cloning techniques. The recent sequencing of the complete genomes of a large number of non-eutherian vertebrates will expedite the isolation and characterization of these immune genes in distantly related species. However, currently automated annotation techniques are not sensitive enough to identify many of these molecules outside the eutherian lineage.

The first marsupial genome was recently sequenced by the Broad Institute. The subject of this project, *Monodelphis domestica*, is a South American opossum. It is a well-recognised biomedical model in the study of comparative physiology, immunogenetics, cancer development and disease susceptibility. Two publicly available annotations of this genome have been generated. Ensembl have produced a gene build with their automatic pipeline [22], which relies principally on GeneWise [23], while the UCSC genome browser provides several annotation tracks with similarity features and gene models, for example chained TBLASTN alignments of human proteins, BLAT alignments of RefSeq mRNAs, and Genscan [24] and N-SCAN [25] predictions. With the exception of the Genscan predictions, which are *ab initio *gene predictions based on genomic sequence only, the gene builds rely on cross species homology, as no large-scale opossum EST projects have been completed yet and there are only 425 known opossum protein sequences in GenBank. In most cases, Ensembl and the UCSC genome browser were unable to identify highly divergent cytokine genes such as IL-2, 4 and 13.

To overcome this shortcoming in the automated annotation of the opossum genome and to start to address uncertainties about immune function in marsupials, we have adopted a manual, expert-curated approach to annotating highly divergent genes. The critical first stage of this is the careful identification of the genomic region containing the gene. This is performed using a sensitive TBLASTN search. HMMER [26] can also be useful at this stage. Frequently, it is necessary to first narrow the search to the syntenic region by identifying conserved flanking genes.

Having identified similarity features, gene prediction is performed on genomic sequence extracted from the region. The accuracy of gene prediction is dependent on the prediction method. As with the automated annotations, we favour gene predictors that incorporate information from orthologous sequences into the prediction process. In addition to GeneWise and N-Scan, there are now several such methods available including Procrustes [27], HMMgene,[28] GenomeScan [29], and Augustus+ [30]. Procrustes and the default GeneWise algorithm perform spliced alignment. Augustus+ uses an interesting approach, which constrains predicted genes to incorporate user-supplied hints. However, it is not particularly convenient for manual use or use by biologists lacking scripting skills. While not the only possible choice, we have found GenomeScan to be both convenient and reasonably accurate (based on comparison with known eutherian sequences). It is worth noting that there is another class of gene prediction methods that obtain homology information from syntenic regions of other genomes. These include TwinScan [31], which is asymmetric and predicts genes in one genome only and SLAM [32], which simultaneously aligns two genomes and predicts genes in both. These methods were unlikely to be useful in our study as we were looking for genes that are highly divergent and difficult to align at the genomic level. Finally, a comparison of predicted results with known eutherian sequences and curation of the result was undertaken if required. Our success with this strategy suggests that this method will be applicable to the identification of rapidly evolving gene families in other distant vertebrate species.

## Results

### Overview

*In silico *searching revealed a total of 23 cytokine sequences, all of which are described in the opossum for the first time and 5 of which are novel for any marsupial species (see Table 1). A number of critical cytokine receptors are also identified, as are the sequences for the hallmark T cell cluster of differentiation markers, CD4 and CD8.

**Table 1:** Comparison of putative opossum and known human cytokine sequences. Opossum IFN-α genes were compared with 13 human IFN-α genes.

Identity	Number of exons in open reading frame		Number of amino acids		% amino acid identity*
**Cytokines**	*opossum*	*human*	*opossum*	*human*	

Interleukin 2	4	4	144	153	41.8
Interleukin 4	4	4	138	153	43.3
Interleukin 5	4	4	137	134	53.0
Interleukin 6	5	5	221	212	36.3
Interleukin 10	5	5	173	178	59.5
Interleukin 12A	7	7	231	253	52.2
Interleukin 13	4	4	122	146	36.7
Interleukin 19	6	6	188	215	58.0
Interleukin 20	6	5	154	176	59.7
Interleukin 21	5	5	163	162	46.5
Interleukin 22	4	5	150	179	40.0
Interleukin 24	6	6	220	206	43.7
Interleukin 26	5	5	201	171	55.9
Interferon γ	4	4	167	166	47.0
*Type 1 Interferons*					
IFN-α (seven genes)	1	1	183	188–9 (range)	33–42 (range)
IFN-β	1	1	184	187	43.2
IFN-κ	1	1	156	207	51.3
**Cytokine receptors**					
Common cytokine receptor gamma chain (IL-2Rγ)	8	8	349	367	54.9
Interferon-γ receptor 2	6	7	269	344	49.6
**T cell surface receptors**					
CD4	9	9	485	458	45.6
CD8	6	6	349	235	37.7

* compared with human sequence

The majority of genes reported in this study were identified using sensitive peptide BLAST searches (Table 2). The most divergent genes, *interleukins 2, 4 *and *13*, were identified using synteny searches. Properties of the putative proteins identified in this study, predicted structures and comparison with human sequences are summarised in Tables 1 and 2. Sequence data of the predicted proteins are available online [33].

**Table 2:** Summary of putative opossum cytokine genes including search strategy, best hit, predicted glycosylation sites and signal peptide information.

Identity	Search Strategy	Best Hit		Number of predicted glycosylation sites		Signal peptide identified
**Cytokines**		*reference*	*e-value*	*N-gly*	*O-gly*	*opossum*

Interleukin 2	synteny	-	-	1	1	Yes
Interleukin 4	synteny	-	-	2	0	Yes
Interleukin 5	BLAST	AAD37462.1	4e-028	1	0	Yes
Interleukin 6	BLAST	NP_112445.1	0.081	2	3	Yes
Interleukin 10	BLAST	AAD01799.1	3e-011	2	0	Yes
Interleukin 12A	BLAST	NP_032377.1	9e-006	1	1	No
Interleukin 13	synteny/HMMER	-	-	0	0	No
Interleukin 19	BLAST	NP_001009940.1	3e-008	3	0	Yes
Interleukin 20	BLAST	NP_061194.2	1e-004	0	0	Yes
Interleukin 21	BLAST	Q9HBE4	0.088	2	1	Yes
Interleukin 22	BLAST	NP_065386.1	3e-005	1	0	Yes
Interleukin 24	BLAST	NP_006841.1	2e-006	2	0	Yes
Interleukin 26	BLAST	NP_060872.1	3e-009	0	0	No
Interferon-γ	synteny	-	-	2	0	Yes
*Type 1 Interferons*						
IFN-α (7 genes)	BLAST	AAO37656.1	1e-019 to 9e-974 (range)	0–4 (range)	0	Yes
IFN-β	BLAST	AAO37656.1		2	0	Yes
IFN-κ	BLAST	AAO37656.1		1	0	No
**Cytokine receptors**						
Common cytokine receptor gamma chain (IL-2Rγ)	BLAST	NP_000197.1	9e-019	6	3	No
Interferon-γ receptor 2	BLAST	NP_005525.2	3e-018	3	2	Yes
**T cell surface receptors**						
CD4	BLAST	NP_000607.1	5e-009	4	0	Yes
CD8	BLAST	Q60965	9e-012	1	8	Yes

### Isolation of interleukins using BLAST and synteny searches

Interleukins 2, 4 and 21 and their common gamma chain receptor were identified using both BLAST and syntenic strategies. IL-21 was identified by a sensitive TBLASTN search (e-value = 2e-18) on Chromosome 5:7034081–7057815. The predicted protein is of similar size and contains the same number of exons as human IL-21 [see Additional File 1]. The signal peptide was predicted to be encoded within the first 21 amino acids (score = 7.6, p = 0.06), with N-linked glycosylation sites predicted at positions 46 and 106 and O-linked glycosylation of threonine predicted at position 55. Instability motifs (ATTTA) were not found in the 3' UTR of the sequence before the poly(A)+ signal.

**Figure 1:**
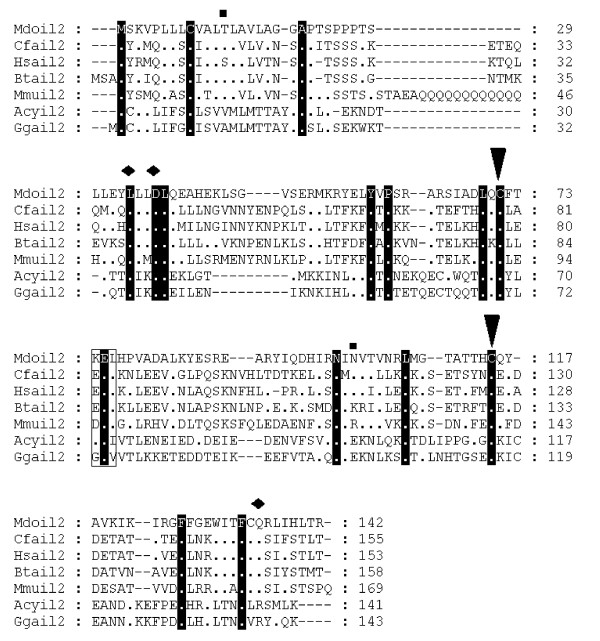
**Alignment of IL-2 amino acid sequences**. Diamonds denote functionally important residues [64]. Inverted triangles indicate cysteine residues involved in disulfide bonds in human protein [64]. Squares above the alignment show predicted glycosylation sites from the opossum sequence. Dots represent identity to *Monodelphis domestica *sequence. Sequences used for alignment: *Homo sapiens *(NP_000577), *Bos taurus *(NP_851340), *Sus scrofa *(NP_999026), *Mus musculus *(NP_032392), *Gallus gallus *(NP_989484), *Canis familiaris *(NP_001003305), *Macaca fascicularis *(Q29615), *Felis cattus *(AAC15974), *Equus caballus *(CAA49190), *Cervus elaphus *(P51747), *Capra hircus *(AAQ10671), *Ovis aries *(NP_001009806), *Oryctolagus cuniculus *(O77620), *Peromyscus maniculatus *(AAP04419), *Rattus norvegicus *(NP_446288), *Anser cygnoides *(AAR28994). Not all sequences are shown in the figure.

Opossum IL-2 was found by searching genomic sequence flanking IL-21, which is adjacent to, and has significant structural homology with IL-2 in humans [see Additional File 2]. This strategy was adopted since the alignment of human IL-2 against the opossum genome using TBLASTN resulted in no hits. A 395 kb region adjacent to IL-21 was extracted and 15 genes within this region were predicted with GENSCAN. The predicted gene most similar to IL-2 was identified using BLASTP. The sequence was extracted and GenomeScan was used with an IL-2 orthologue to obtain a more accurate prediction. Opossum IL-2 was located on Chromosome 5:7191593–7196834 (Fig 1) and contains several conserved residues essential for biological activities, including two cysteine residues that provide structural stability [34] and the amino acids leucine and aspartic acid within helix A, which are crucial for binding of the ligand to IL-2Rβ in humans [35]. Also well conserved is a glutamine residue in the D helix, which is directly involved in the binding of the IL-2Rγ chain [36]. Similar to the human sequence, the putative peptide is 142 amino acids in length and contains 4 exons. A signal peptide that contains a potential O-linked glycosylation site (position 13 – Thr) is predicted from positions 1–22 (score = 9.9, p = 0.03). A potential N-linked glycosylation site, not found in humans or mice, but present in several eutherians including the cat and dog, is found at position 101. Four mRNA instability motifs (ATTTA) are present upstream of the poly(A)+ signal.

Opossum IL-2Rγ was identified using TBLASTN (e-value = 8e-119) (Table 1). It shares 61% amino acid similarity with the human sequence [see Additional File 3].

IL-5 was identified on chromosome 1:307529660–307531352. It shares 53.0% identity to human IL-5, and 86.7% identity to the tammar wallaby IL-5 [13] [see Additional File 4].

**Figure 2:**
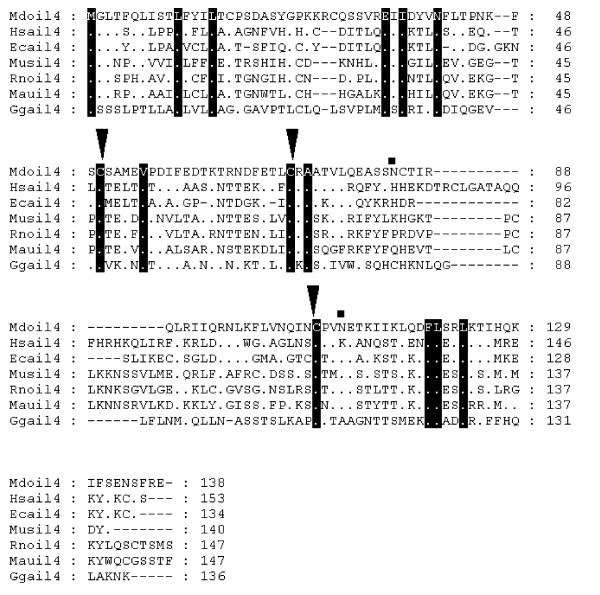
**Alignment of IL-4 amino acid sequences**. Inverted triangles indicate cysteine residues that form disulfide bonds in the human protein [65]. Squares above the alignment show predicted N-linked glycosylation sites in the opossum sequence. Dots represent identity to *Monodelphis domestica *sequence. Sequences used for alignment: *Homo sapiens *(NP_000580), *Mus musculus *(NP_067258), *Gallus gallus *(NP_001007080), *Equus caballus *(P42202), *Rattus norvegicus *(NP_958427), *Mesocricetus auratus *(Q60440).

Synteny searches located the sequence for *IL-4 *[see Additional File 5]. *RAD50 *(GenBank accession no: AAB07119) and kinesin-like protein *KIF3A *(GenBank accession no: NP_008985) are situated adjacent to *IL-4 *and *IL-13 *in humans. The area between these proteins in opossum was extracted and GENSCAN predictions were searched with BLASTP and FASTP for suitable matches. *IL-4 *was identified using FASTP and was located on Chromosome 1 (307752915–307754456). The predicted peptide is 138 amino acids in length (Fig 2). It has low levels of identity to human *IL-4 *(30.8%). Two putative N-linked glycosylation sites were identified. SPScan was unable to predict a putative signal sequence although two instability motifs (ATTTA) were recognised in the 3' UTR region. Despite the variation in sequence between the predicted opossum and human IL-4 protein sequences, disulfide bonds that join helix B to the CD loop and that are important for biological activity are conserved [37].

**Figure 3:**
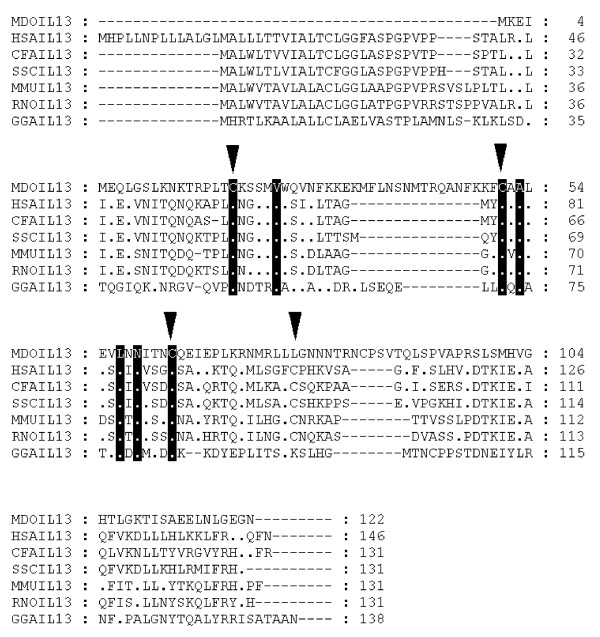
**Alignment of IL-13 amino acid sequences**. Inverted triangles indicate cysteine residues that form disulfide bonds in the human protein [66]. Dots represent identity to *Monodelphis domestica *sequence. Sequences used for alignment: *Homo sapiens *(NP_002179), *Mus musculus *(NP_032381), *Gallus gallus *(NP_001007086), *Rattus norvegicus *(NP_446280), *Canis familiaris *(NP_001003384), *Sus scrofa *(Q95J68).

*IL-4 *and *IL-13 *were identified simultaneously using a syntenic approach since they sit adjacent in the human genome [see Additional File 5]. Opossum *IL-13 *(Chromosome 1:307682382–307686155) is found 74.30 kb upstream from opossum *IL-4 *and does not contain any glycosylation sites. Alignment with mammal and chicken protein sequences (Fig 3) revealed a truncation of 32 amino acids from the 5' end of the peptide in opossum *IL-13*. This is probably due to incorrect gene prediction, a fact supported by the absence of signal peptide and any instability motifs.

Opossum *IL-6 *was identified using a sensitive TBLASTN search (e-value = 0.08). Opossum IL-6 is located on Chromosome 8:296810942–296824133 and the PROSITE IL-6 family motif (C-x(9)-C-x(6)-G-L-x(2)- [F,Y]-x(3)-L) is conserved [see Additional File 6]. The signal peptide is predicted from positions 1–28 (score = 8.1, p = 0.20) and no instability motifs (ATTTA) are found in the 3' UTR. Opossum IL-6 has maintained significant structural similarities to human and other mammalian IL-6 genes despite its comparatively low sequence identity. The number and position of cysteine residues in opossum IL-6 are identical to those found in eutherian and chicken sequences. An arginine molecule in helix D that is involved in IL-6β binding [38] is also conserved.

Opossum IL-12 alpha chain (chr7:260,616,009–260,626,803) was identified using a TBLASTN search and is predicted to be 58% similar to its human orthologue [see Additional File 7]. Cysteine residues are conserved between the marsupial, eutherians and chicken sequences.

IL-10 family members were identified in two clusters. Chromosome 2 contained *IL-10 *(113139397–113144942; [see Additional File 8]), *IL-19 *(113283404–113294773; [see Additional File 9]), *IL-20 *(113319666–113324608; [see Additional File 10]), *IL-24 *(113362216–113377467; [see Additional File 11]) with identical head-to-tail transcriptional orientation and organisation to their human orthologues. Chromosome 8 contained *IL-26 *(23485674–23494985; [see Additional File 12]) and *IL-22 *(23457582–23460076; [see Additional File 13]). The complete *IL-22 *open reading frame was not identified since the 3' end (approximately 33 amino acids and 2 exons) fell in an unsequenced gap. However, conservation of a predicted N-linked glycosylation site at N54 between putative opossum *IL-22 *and human *IL-22 *(a site crucial for IL-22 modulation during the inflammatory response) suggests that this partial sequence is opossum *IL-22*. Both chicken and the amphibia contain IL-10 family members, although only one IL-19-like ancestral gene replaces IL-19, IL-20 and IL-24 in the chicken [39]. Orthology of the IL-10 family cytokines with their eutherian counterparts was confirmed by phylogenetic analysis [see Additional File 14]. All putative IL-10 family members clustered closely with their eutherian orthologs.

### Isolation of cluster of differentiation markers using TBLASTN

CD4 [see Additional File 15] and CD8 [see Additional File 16] were identified by TBLASTN search and found on chromosome 8 (104157682–104183462) and chromosome 1 (716671734–716675645) respectively. Their number of amino acids and potential glycosylation sites are noted in Table 2. Neither we, nor Ensembl, were able to successfully predict the terminal exons of these two genes.

### Isolation of interferons using BLAST, synteny and hidden Markov models

#### Type I IFNs

**Figure 4:**
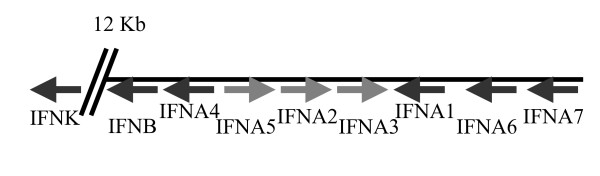
Genomic organisation and transcriptional directions of type I IFNs on chromosome 6.

**Figure 5:**
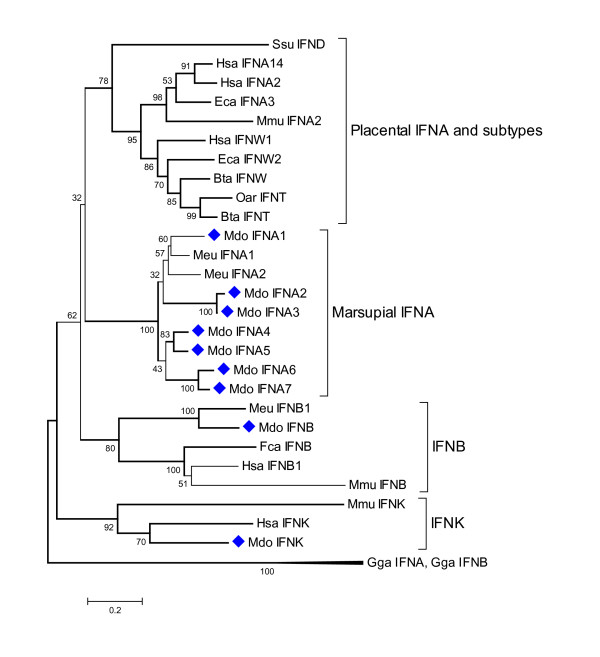
**Phylogenetic tree showing evolutionary relationship between type I interferon protein sequences**. Opossum sequences are marked by diamonds. Sequences used: *Homo sapiens *IFN-α14 (NP_002163.1), IFN-α2 (NP_000596.2), IFN-β (NP_002167), IFN-ω1 (P07352), IFN-κ(NP_064509.1); *Mus musculus *IFN-α2 (P01573), IFN-β (NP_034640), IFN-κ (NP_954608.1); *Sus scrofa *IFN-δ (NP_001002832.1); *Equus caballus *IFN-α3 (CAA01292), IFN-ω2 (CAA01293); *Bos taurus *IFN-ω1 (P07352), IFN-t (XP_874910); *Ovis aries *IFN-τ(CAA39783); *Felis catus *IFN-β (Q9N2J0); *Macropus eugenii *IFN-α1 (AAO37656), IFN-α2 (AAO37657.1), IFN-β (AAO37658.1); *Gallus gallus *IFN-α1 (CAA63214), IFN-β (NP_001020007).

Nine type I IFN coding sequences and 2 pseudogenes were identified in the opossum genome using BLAST strategies. Seven IFN-α genes, along with single copies of IFN-β and IFN-κ were identified. Predicted opossum IFN-α sequences share 68–78% identity and 78–99% similarity at the amino acid level. IFN-α and -β genes were located in a cluster on Chromosome 6, with IFN-κ situated approximately 12 kb away (Fig 4). Phylogenetic analysis revealed that opossum IFN-α sequences were interspersed with known tammar wallaby IFN-αs (Fig 5).

#### Interferon gamma (IFN-γ) and interferon gamma receptor (IFN-γR2)

**Figure 6:**
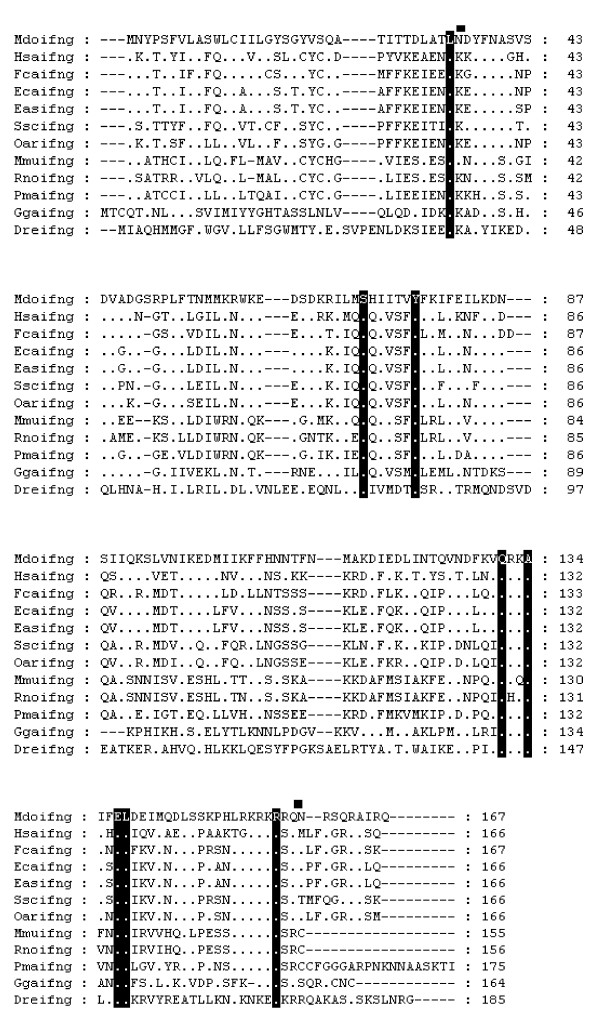
**Alignment of IFN-γ amino acid sequences**. Squares above the alignment show predicted N-linked glycosylation sites from the opossum sequence. Dots represent identity to *Monodelphis domestica *sequence. Sequences used for alignment: *Homo sapiens *(NP_000610.2), *Felis cattus *(P46402), *Equus caballus *(P42160), *Equus asinus *(O77763), *Sus scofa *(NP_999113.1), *Ovis aries *(P17773), *Mus musculus *(NP_032363.1), *Rattus norvegicus *(NP_620235.1), *Peromyscus maniculatus *(AAP44086.1), *Gallus gallus *(P49708), *Danio rerio *(NP_998029.1).

The signal transducing chain of the Interferon gamma receptor was identified in the opossum genome on Chromosome 4 (14328267–14355149). It shares 29–46% amino acid identity with eutherian and chicken sequences [see Additional File 17]. The ligand of IFN-γR2, IFN-γ, was not identified in the genome, despite exhaustive searches including searches using the Hidden Markov model (HMM) containing predicted ancestral sequences. According to the gene organization in other vertebrates (including birds and fish), IFN-γ should be adjacent to IL-22 and IL-26 on chromosome 8. A large gap (9.6 kb) was located in this region, suggesting that IFN-γ was simply not sequenced, rather than being absent from the genome. However, the availability of BAC end sequences generated by the genome sequencing project did allow us to identify a BAC (VMRC-18:653P7) that spanned this region. Researchers at the Broad Institute, led by Kerstin Lindblad-Toh and April Cook kindly sequenced this BAC (GenBank Accession: AC190119). IFN-γ was thus identified (Fig 6). It shares 47% amino acid identity with human IFN-γ but predicted glycosylation sites are unique.

### Level of confidence in our gene predictions

Where possible, gene predictions were verified by alignment with known marsupial cDNA sequences, and compared to Ensembl gene predictions and UCSC similarity features. For instance, a known cDNA sequence is available for *Trichosurus vulpecula *(possum) IL-10 cDNA (Genbank ref: AF026277). Our predicted opossum IL-10 protein shared 76% amino acid identity with possum IL-10, and exon-intron boundaries match. However, despite our use of robust methodologies, we are still not confident with prediction of the most divergent immune genes sequences. Some doubt exists with our predications for IL-4 and IL-13 and the terminal exons of IL-22, CD4 and CD8. Characterisation of their cDNA, together with laboratory-based assays will ultimately confirm the reliability of the predictions reported here.

## Discussion

Without EST and protein databases, annotation of distantly related mammalian species such as the marsupials and monotremes is challenging. Neither Ensembl nor UCSC were able to identify IL-2, 4, 13, 22 and IFN-γ. In general, automated gene prediction missed key immune genes because of their low levels of sequence similarity with their eutherian orthologs. We suggest that future studies focusing on *in silico *mining of divergent genes should take into account gene location and features. Application of this strategy allowed us to successfully identify key immune genes in the opossum genome, which traditional laboratory methods failed to isolate.

Discovery of key cytokines in the opossum genome suggests that a re-examination of immune responses (especially T cell responses) is warranted in marsupials. The peculiarities in class switching and *in vitro *T cell proliferation, which have previously been observed in marsupials are largely controlled by T cells and their products. The ability to discriminate between classic 'helper' T cells and 'cytotoxic' T cell families will now be possible due to the identification of CD4 and CD8 sequences in the opossum genome. Further, identification of cytokines normally produced by these subsets in eutherian mammals will allow us to investigate Th1 and Th2 profiles that orchestrate immunity to intracellular and extracellular pathogens respectively.

There are a myriad of interactions between cytokines at the cellular level, but the presence of a number of key cytokines orchestrate the global immune response. For example, when the macrophage-derived IL-12 is dominant, Th1 responses predominate resulting in cell-mediated immunity. When the B-cell growth factor IL-4 is dominant, Th2 responses dominate and a humoral immune response is activated [40]. Sequences for both of these genes are present in the opossum genome, along with other classical Th1- (IL-2, IFN-γ) and Th2- (IL-4, IL-5, IL-6, IL-10 and IL-13) associated molecules.

The presence of key cytokines in a marsupial genome strongly suggests that marsupials are capable of complex immune responses comparable to those seen in eutherian mammals. Knowledge of these gene sequences provides a springboard for future studies. For instance, marsupials appear to be susceptible to infection with intracellular pathogens such as herpesvirus and mycobacterial spp [41], indicative of impaired Th1 cytokine responses. The availability of Th1 and Th2 cytokine sequences will allow us to study IL-10 profiles, which are known to play a critical role in the survival of intracellular pathogens by inhibiting the expression of inflammatory cytokines such as IFN-γ and TNF. Meanwhile, studies of Th2 cytokines may focus on protection against parasites. Both American and Australian marsupials co-exist with a range of successful parasites; opossums are reported to have natural trypanosome infection rates of up to 100% [42] and carry nematode burdens in the wild [43], whilst a variety of helminth infections are common across a range of Australian marsupials [44].

Allograft responses can now be studied due to the availability of sequence information for interleukins 2, 4, 21 and IL-2Rγ [45]. The opossum is an important model for tumour immunology since it can be induced to accept melanoma cells at both juvenile and adult life stages [46]. Both IL-2 and IL-24 are associated with melanoma tumour suppression [47] in humans and it is now possible to study the role of these genes in the opossum model, as well as in the maintenance of transmissible allograft tumours in Tasmanian devil facial tumour disease [48].

## Conclusion

Here we describe and apply a method to identify divergent immune genes from the genome of a model marsupial, *Monodelphis domestica*. We are now extending this analysis to characterize the entire opossum immunome. We report here that the opossum genome contains representatives from the major vertebrate immune gene families. These genes appear to be structurally similar, and therefore will most likely prove to be functionally equivalent, to their eutherian homologues. The way is now clear to further probe the genes that orchestrate the marsupial immune response and to investigate the role that these molecules have on maintaining health and influencing disease susceptibility in this unique group of animals.

## Methods

### Data source

Draft sequencing of the genome of a female opossum (*Monodelphis domestica*) has recently been completed by the Broad Institute [49]. Analysis was performed on assembly MonDom4 (January 2006).

### Sequence identification

To optimise the chances of identifying previously undiscovered sequences, our search strategy relied on a preliminary database screen for sequence conservation, together with positional analyses of the gene sequence relative to other genes within the genome (synteny). Finally, the putative sequence was analysed for the presence of biologically significant sites associated with both structure and function in their eutherian homologues.

### Similarity searching using BLAST

Sequence similarity searching (TBLASTN) was performed with known eutherian sequences. Positive hits from the BLAST search with good potential were extracted for further structural analysis. When ambiguities existed between alignments from BLAST results, each of the multiple hits were extracted and inspected. Assessment methods for ambiguous hits included tests for reciprocal-best-hit where the aligned sequence was blasted against SWISS-PROT and TrEMBL protein databases to confirm preliminary findings. Proteins discovered in BLAST searches were used to mine additional homologues. To do this, parameters were optimised for sensitive searching. In order to increase our ability to detect highly divergent sequences, the BLOSUM 45 similarity matrix [50] was used. Additionally, application of soft-masking and the lowering of the neighbourhood word threshold score to 9 increased the chance of detecting homologous sequences that otherwise might have been overlooked using default parameters.

### Synteny analysis

If the protein of interest was not detected by the initial BLAST search, other methods were employed. Similarity searches were performed with genes found in close syntenic regions in the human genome. Syntenic regions were extracted from the opossum database, and passed into GENSCAN [24]. The predicted peptide sequences were analysed by performing similarity searches against the SWISS-PROT and TrEMBL databases using BLASTP and FASTP [51]. In order to improve the accuracy of identified cytokine sequences, the sequence was re-extracted from the opossum database and the putative protein was re-evaluated using GenomeScan [29]. Combined results from GenomeScan and GENSCAN were compared with documented structural features of the cytokine.

### Additional methods for gene identification

For sequences that were not detected using the above methods, a hidden Markov model (HMM) was built and calibrated using the HMMER 2.3.2 package [52]. The model was built as a multiple local alignment profile with the Krogh/Mitchison substitution weight matrix [53] and used to search the six-frame translation of the opossum genome.

Ancestral sequences were included in the HMM. These were calculated by programs in the Phylip package [54]. PRODIST was used to compute a distance matrix under default settings. After this, the program NEIGHBOUR was used to create a neighbour joining (NJ) tree from the matrix. The tree was rooted with a teleost species. Following this, ProML was set to produce ancestral sequences at each of the NJ tree nodes.

### Structural features

Once the gene of interest was located, exon/intron boundaries were identified using the gene prediction programs GENSCAN [24] and GenomeScan [29]. Our experience suggests that some caution is advisable in the interpretation of data from existing gene prediction software; excessively long predicted genes ('thready' gene predictions) due to mis-identification of first exons and merging of adjacent genes, and unlikely predictions of splice sites (based on comparison with orthologous sequences) were the most common problems we observed. Mindful of these limitations, our gene predictions were compared with known gene structures. The presence of signal peptides was predicted by SPScan (Accelrys GCG) and estimation of glycosylation sites were made with NetOGlyc 3.1 [55] and NetNGlyc 1.0 [56]. Finally, sequences were submitted to the PROSITE database [57] for detection of protein family motifs that would confirm gene identify.

### Sequence alignments

Sequences from the opossum and other species were aligned using ClustalW [58]. Accession numbers of sequences used in analyses are shown in figure legends. Sequence labels in the alignments are abbreviated by the first letter from the genus with the first two letters from the species name followed by the gene name. In figures, residues with functional importance are highlighted.

### Phylogenetic analysis

Neighbour-joining (NJ) trees were constructed using the Jones-Taylor-Thornton substitution model [59] and 500 bootstrap replicates in MEGA 3.1 [60]. The tree, constructed from amino acid sequences, was rooted using chicken sequences.

### Sequence identity

Sequence identity and similarity calculations were carried out using GAP (Accelrys GCG), with the Needleman-Wunsch alignment [61], except for IFN-α genes which were calculated in GenDoc [62] using the BLOSUM 35 similarity matrix [50] for comparisons of human and opossum IFN-α genes and BLOSUM 80 matrix [50] for comparisons among opossum sequences. GCG, GENSCAN, BLASTP and FastA programs were accessed through the Australian National Genomic Information Service (ANGIS) [63].

## Competing interests

The author(s) declare that they have no competing interests.

## Authors' contributions

EW performed the bioinformatics studies and helped to draft the manuscript

LJY wrote the final manuscript and participated in the design of the study

ATP critically revised the bioinformatics data and co-ordinated the design of the bioinformatics approach

KB conceived of the study and co-ordinated and helped with the preparation of the final manuscript

All authors read and approved the final manuscript

## Supplementary Material

Additional File 1 **Alignment of IL-21 amino acid sequences**. Squares above the alignment show predicted glycosylation sites from the opossum sequence. Residues Asp33 and Gln145 are important for receptor binding in humans and are denoted by a diamond [71]. Inverted triangles indicate cysteine residues that are conserved across species. Dots represent identity to *Monodelphis domestica *sequence. Sequences used for alignment: *Homo sapiens *(Q9HBE4), *Mus musculus *(NP_068554.1), *Gallus gallus *(NP_001020006.1), *Canis familiaris *(NP_001003347.1), *Sus scofa *(Q76LU6), *Bos taurus *(Q76LU5). Click here for file

Additional File 2 **Syntenic region between human chromosome 4q27 and opossum chromosome 5, illustrating the gene cluster of interleukin 2 and 21**. Transcriptional directions are indicated by arrows. Click here for file

Additional file 3 **IL-2Rγ amino acid sequences**. Conserved cysteine residues are marked with an inverted triangle. Dots represent identity to *Monodelphis domestica *sequence. Completely conserved residues are shaded. Sequences used for alignment: *Homo sapiens *(NP_000197.1), *Mus musculus *(NP_038591.1), *Gallus gallus *(NP_989858.1), *Rattus norvegicus *(NP_543165.1), *Canis familiaris *(NP_001003201.1), *Sus scrofa *(NP_999248.1), *Bos taurus *(NP_776784.1). Click here for file

Additional file 4 **Alignment of IL-5 amino acid sequences**. Dots represent identity to *Monodelphis domestica *sequence. Sequences used for alignment: *Homo sapiens *(NP_000870.1), *Macaca mulatta *(NP_001040598.1), *Bos taurus *(NP_776347.1), *Canis familiaris *(NP_001006951.1), *Mus musculus *(NP_034688.1), *Macropus eugenii *(AAD37462.1), *Gallus gallus *(NP_001007085.1). Click here for file

Additional file 5 **Syntenic region between human chromosome 5q23.3 and opossum chromosome 1, illustrating the gene cluster of interleukin 5, 4 and 13**. Transcriptional directions are indicated by arrows. Click here for file

Additional file 6 **Alignment of IL-6 amino acid sequences**. Residues involved in receptor binding in human IL-6 are denoted with diamonds. Cysteine residues conserved among all species are marked with an inverted triangle. PROSITE family motif is boxed. Dots represent identity to *Monodelphis domestica *sequence. Sequences used for alignment: *Homo sapiens *(NP_000591.1), *Mus musculus *(NP_112445.1), *Oryctolagus cuniculus *(Q9MZR1). Click here for file

Additional file 7 **Alignment of IL-12α amino acid sequences**. Cysteine residues conserved among all species are marked with an inverted triangle. Dots represent identity to *Monodelphis domestica *sequence. Sequences used for alignment: *Homo sapiens *(NP_000873.2), *Mus musculus *(NP_032377.1), *Gallus gallus *(NP_998753.1), *Rattus norvegicus *(NP_445842.1), *Ovis aries *(NP_001009736.1) *Canis familiaris *(NP_001003293.1). Click here for file

Additional file 8 **Alignment of IL-10 amino acid sequences**. Dots indicate identity to *Monodelphis domestica *sequence. Sequences used for alignment: *Homo sapiens *(NP_000563.1), *Mus musculus *(NP_034678.1), *Gallus gallus *(NP_001004414.1), *Trichosurus vulpecular *(AAD01799), *Canis familiaris *(NP_001003077.1), *Sus scofa *(Q29055), *Cervus elaphus*(P51746). Click here for file

Additional file 9 **Alignment of IL-19 amino acid sequences**. Dots indicate identity to *Monodelphis domestica *sequence. Sequences used for alignment: *Homo sapiens *(NP_037503.2), *Mus musculus *(NP_001009940.1). Click here for file

Additional file 10 **Alignment of IL-20 amino acid sequences**. Dots indicate identity to *Monodelphis domestica *sequence. Sequences used for alignment: *Homo sapiens *(NP_061194.2), *Mus musculus *(NP_067355.1), *Tetraodon nigroviridis *(AAP57416.1). Click here for file

Additional file 11 **Alignment of IL-24 amino acid sequences**. Dots indicate identity to *Monodelphis domestica *sequence. Sequences used for alignment: *Homo sapiens *(NP_006841.1), *Mus musculus *(NP_444325.1), *Rattus norvegicus *(NP_579845.1), *Tetraodon nigroviridis *(AAP57418.1). Click here for file

Additional file 12 **Alignment of IL-26 amino acid sequences**. Dots indicate identity to *Monodelphis domestica *sequence. Sequences used for alignment: *Homo sapiens *(NP_060872.1), *Danio rerio *(NP_001018635.1). Click here for file

Additional file 13 **Alignment of IL-22 amino acid sequences**. Dots indicate identity to *Monodelphis domestica *sequence. Sequences used for alignment: *Homo sapiens *(NP_065386.1), *Mus musculus *(NP_058667.1), *Sus scofa *(AAX33671.1), *Rattus norvegicus *(ABF82262.1), *Danio rerio *(NP_001018628.1). Click here for file

Additional file 14 **Neighbour-Joining tree of IL-10 family ligand protein sequences rooted by midpoint**. JTT amino acid substitution matrix was used and 500 bootstrap replicates performed. Branches supported by bootstrap values over 70 are in bold. Opossum sequences are marked by triangles. Sequences used for this analysis were *Homo sapiens *IL-10 (NP_000563.1), IL-19 (NP_715639.1), IL-20 (NP_061194.2), IL-22 (NP_065386.1), IL-24 (NP_006841.1), IL-26 (NP_060872.1); *Mus musculus *IL-10 (NP_034678.1), IL-19 (NP_001009940.1), IL-20 (NP_067355.1), IL-22 (NP_058667.1), IL-24 (NP_444325.1); *Rattus norvegicus *IL-24 (NP_579845.1); *Sus scofa *IL-10 (Q29055); *Bos taurus *IL-10 (P43480); *Trichosurus vulpecular *IL-10 (AAD01799); *Gallus gallus *IL-10 (NP_001004414.1); *Cyprinus carpio *IL-10 (BAC76885.1); *Tetraodon nigroviridis *IL-10 (CAD67786.1); *Takifugu rub*ripes IL-10 (CAD62446.1) *Danio rerio *IL-26 (NP_001018635.1) and *Monodelphis domestica*. Sequences labels in the tree are abbreviated by the first letter from the genus with the first two letters from the specific name followed by the gene name. Click here for file

Additional file 15 **Alignment of CD4 amino acid sequences**. Dots indicate identity to *Monodelphis domestica *sequence. Sequences used for alignment: *Homo sapiens *(NP_000607.1), *Mus musculus *(NP_038516.1), *Macaca mulatta *(BAA09671.1) *Felis cattus *(NP_001009250.1), *Rattus norvegicus *(NP_036837.1) *Oncorhynchus mykiss *(AAY42068.1). Click here for file

Additional file 16 **Alignment of CD8 amino acid sequences**. Dots indicate identity to *Monodelphis domestica *sequence. Sequences used for alignment: *Homo sapiens *(NP_001759.3), *Mus musculus *(Q60965), *Gallus gallus *(NP_990566.1), *Canis familiaris *(NP_001002935.1), *Sus scofa *(NP_001001907.1), *Rattus norvegicus *(AAH88126.1). Click here for file

Additional file 17 **Alignment of IFNGR-2 amino acid sequences**. Cysteine residues conserved among all species are marked with an inverted triangle. Dots indicate identity to *Monodelphis domestica *sequence. Sequences used for alignment: *Homo sapiens *(NP_005525.2), *Mus musculus *(NP_032364.1), *Gallus gallus *(NP_001008676.1). Click here for file
