# Supplementary figures and images for: In silico identification of opossum cytokine genes suggests the complexity of the marsupial immune system rivals that of eutherian mammals
Source: Immunome Res. 2006 Nov 10;2:4. doi: 10.1186/1745-7580-2-4 (PMC1660534; doi:10.1186/1745-7580-2-4)

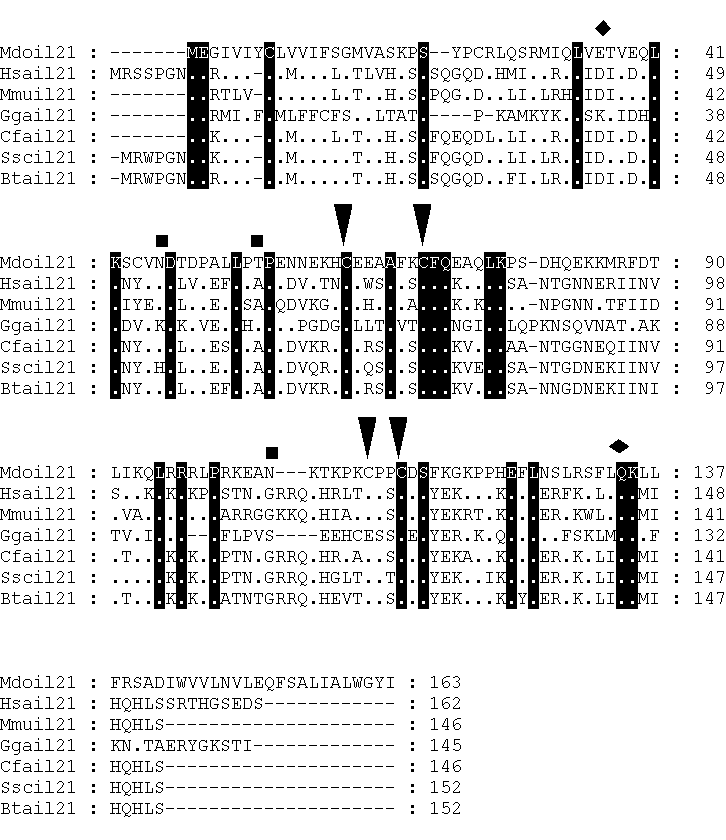

Supplement: Additional File 1 — Alignment of IL-21 amino acid sequences. Squares above the alignment show predicted glycosylation sites from the opossum sequence. Residues Asp33 and Gln145 are important for receptor binding in humans and are denoted by a diamond [71]. Inverted triangles indicate cysteine residues that are conserved across species. Dots represent identity to Monodelphis domestica sequence. Sequences used for alignment: Homo sapiens (Q9HBE4), Mus musculus (NP_068554.1), Gallus gallus (NP_001020006.1), Canis familiaris (NP_001003347.1), Sus scofa (Q76LU6), Bos taurus (Q76LU5). [file 1745-7580-2-4-S1.doc]

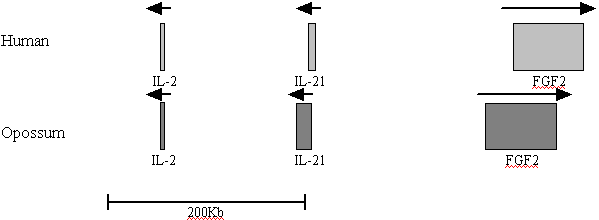

Supplement: Additional File 2 — Syntenic region between human chromosome 4q27 and opossum chromosome 5, illustrating the gene cluster of interleukin 2 and 21. Transcriptional directions are indicated by arrows. [file 1745-7580-2-4-S2.doc]

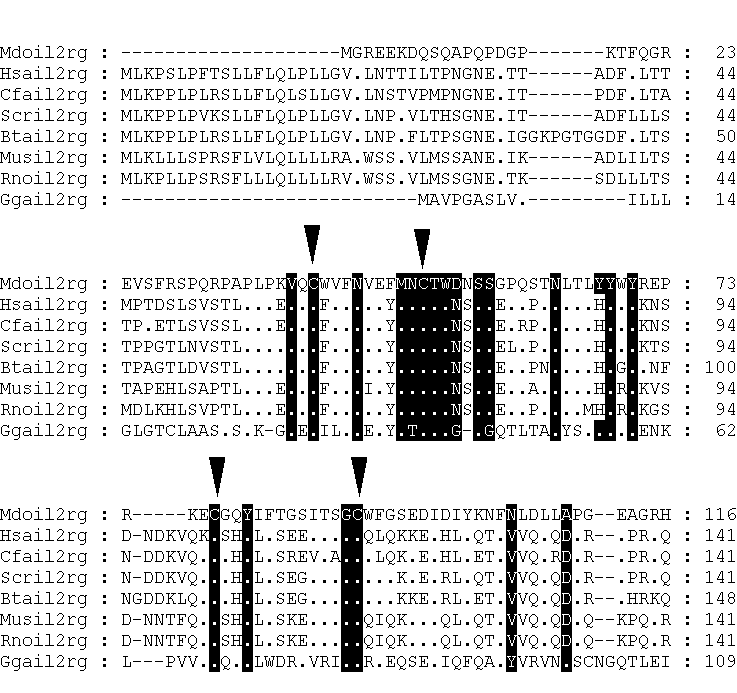


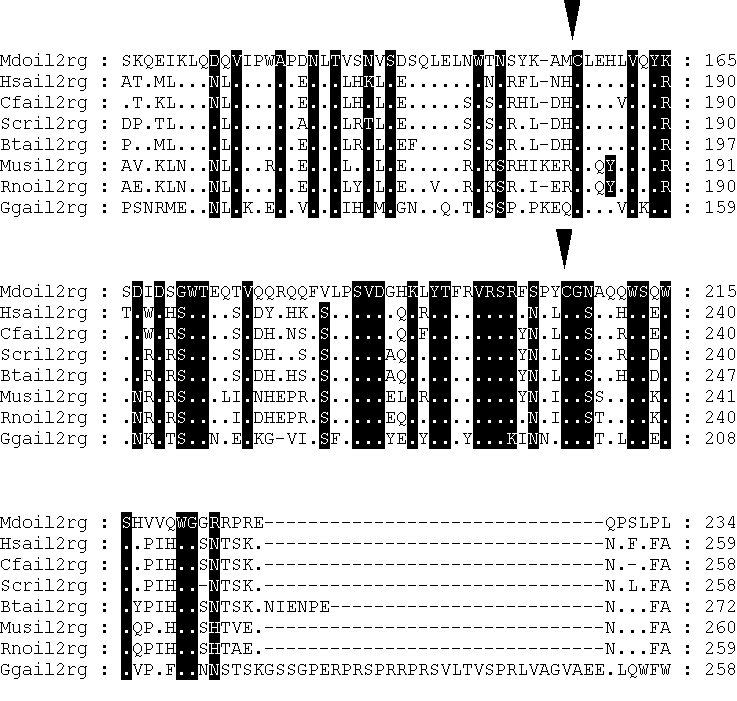


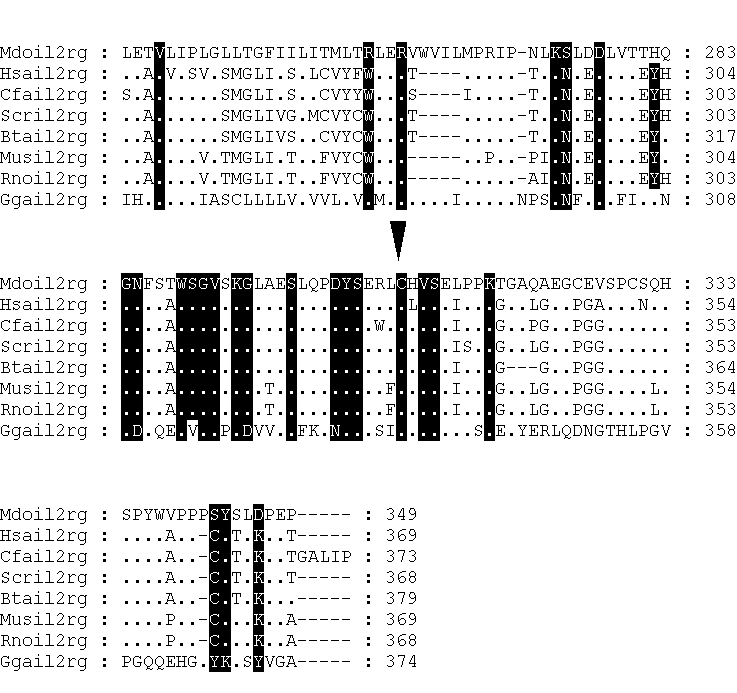

Supplement: Additional file 3 — IL-2Rγ amino acid sequences. Conserved cysteine residues are marked with an inverted triangle. Dots represent identity to Monodelphis domestica sequence. Completely conserved residues are shaded. Sequences used for alignment: Homo sapiens (NP_000197.1), Mus musculus (NP_038591.1), Gallus gallus (NP_989858.1), Rattus norvegicus (NP_543165.1), Canis familiaris (NP_001003201.1), Sus scrofa (NP_999248.1), Bos taurus (NP_776784.1). [file 1745-7580-2-4-S3.doc]

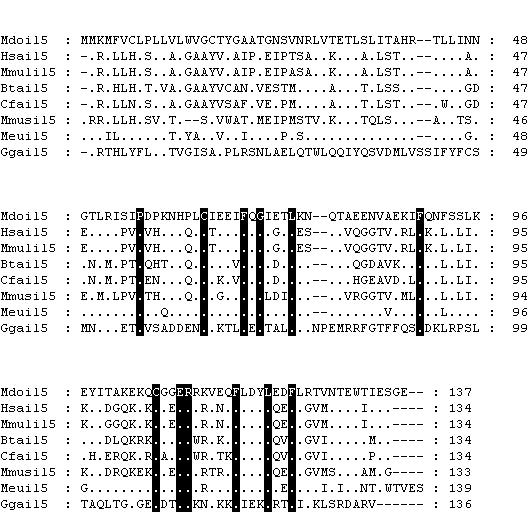

Supplement: Additional file 4 — Alignment of IL-5 amino acid sequences. Dots represent identity to Monodelphis domestica sequence. Sequences used for alignment: Homo sapiens (NP_000870.1), Macaca mulatta (NP_001040598.1), Bos taurus (NP_776347.1), Canis familiaris (NP_001006951.1), Mus musculus (NP_034688.1), Macropus eugenii (AAD37462.1), Gallus gallus (NP_001007085.1). [file 1745-7580-2-4-S4.doc]

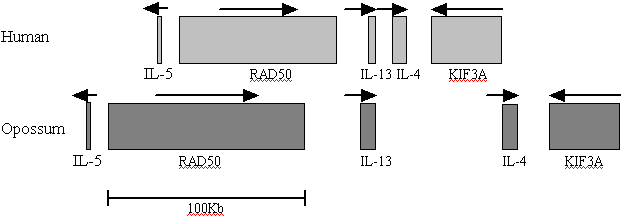

Supplement: Additional file 5 — Syntenic region between human chromosome 5q23.3 and opossum chromosome 1, illustrating the gene cluster of interleukin 5, 4 and 13. Transcriptional directions are indicated by arrows. [file 1745-7580-2-4-S5.doc]

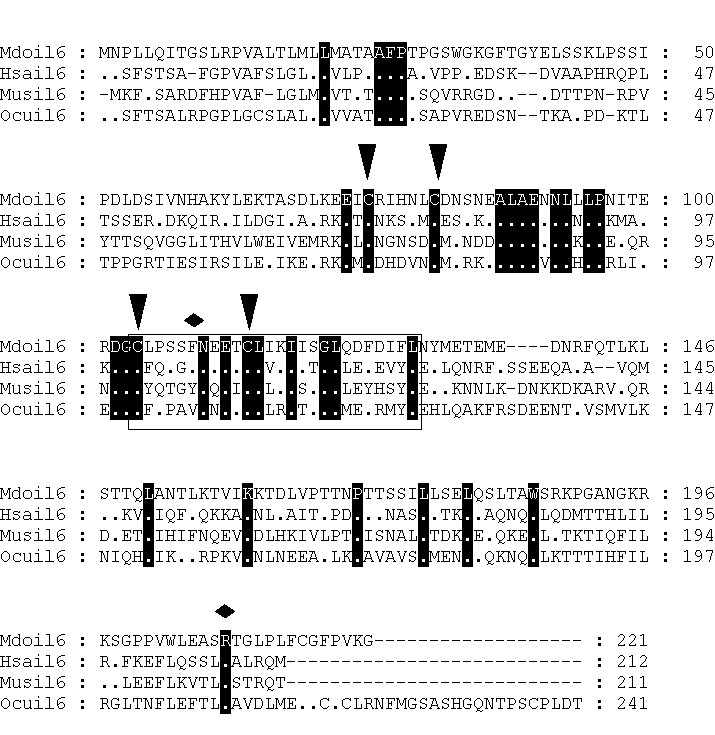

Supplement: Additional file 6 — Alignment of IL-6 amino acid sequences. Residues involved in receptor binding in human IL-6 are denoted with diamonds. Cysteine residues conserved among all species are marked with an inverted triangle. PROSITE family motif is boxed. Dots represent identity to Monodelphis domestica sequence. Sequences used for alignment: Homo sapiens (NP_000591.1), Mus musculus (NP_112445.1), Oryctolagus cuniculus (Q9MZR1). [file 1745-7580-2-4-S6.doc]

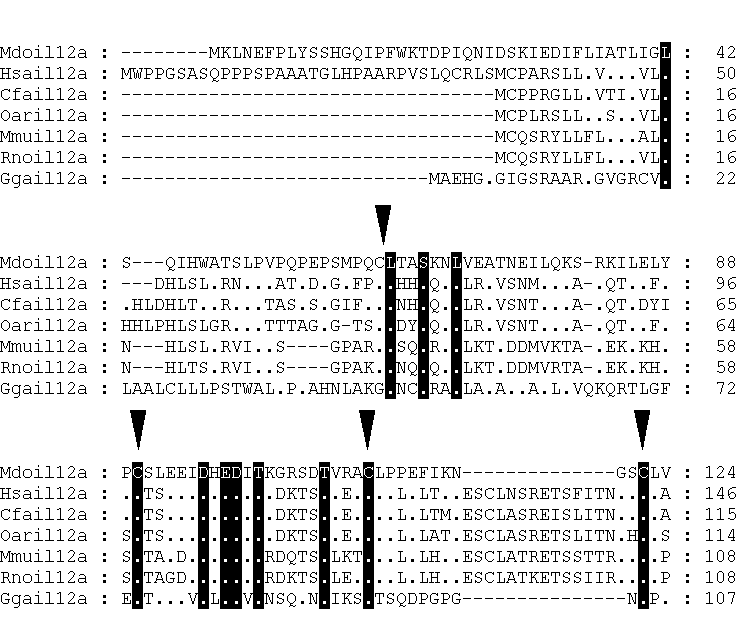


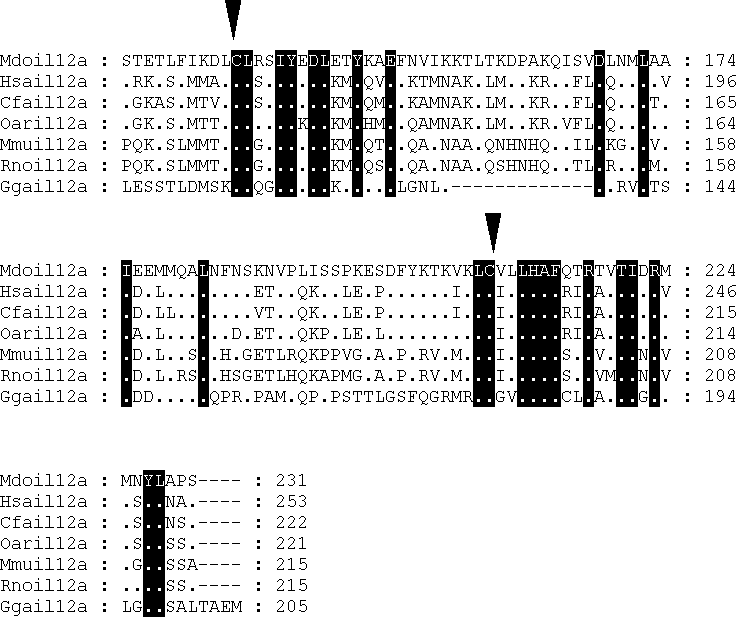

Supplement: Additional file 7 — Alignment of IL-12α amino acid sequences. Cysteine residues conserved among all species are marked with an inverted triangle. Dots represent identity to Monodelphis domestica sequence. Sequences used for alignment: Homo sapiens (NP_000873.2), Mus musculus (NP_032377.1), Gallus gallus (NP_998753.1), Rattus norvegicus (NP_445842.1), Ovis aries (NP_001009736.1) Canis familiaris (NP_001003293.1). [file 1745-7580-2-4-S7.doc]

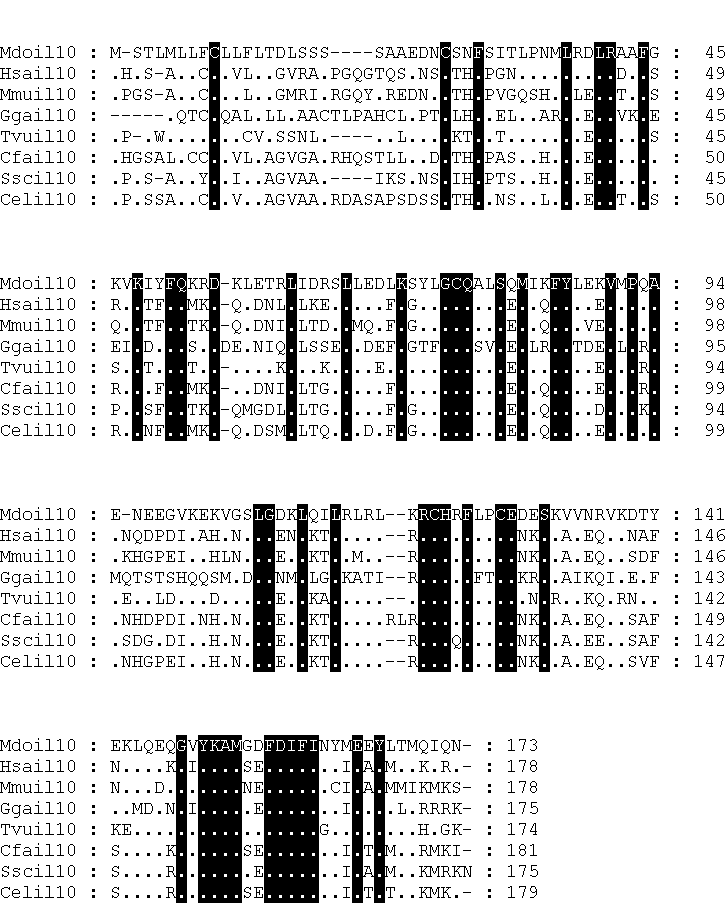

Supplement: Additional file 8 — Alignment of IL-10 amino acid sequences. Dots indicate identity to Monodelphis domestica sequence. Sequences used for alignment: Homo sapiens (NP_000563.1), Mus musculus (NP_034678.1), Gallus gallus (NP_001004414.1), Trichosurus vulpecular (AAD01799), Canis familiaris (NP_001003077.1), Sus scofa (Q29055), Cervus elaphus(P51746). [file 1745-7580-2-4-S8.doc]

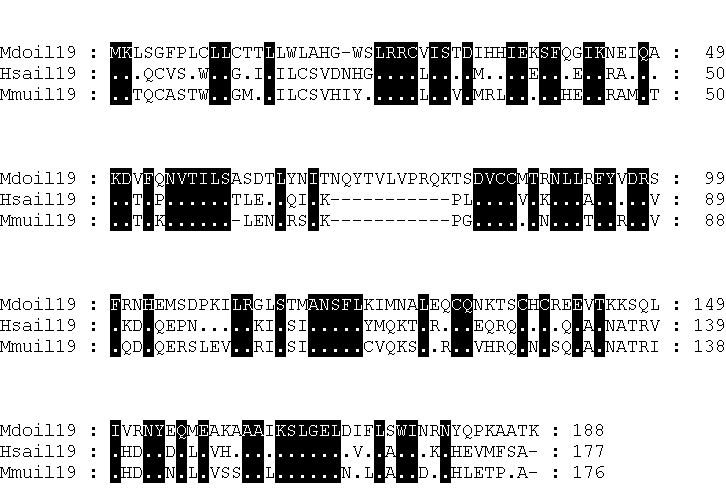

Supplement: Additional file 9 — Alignment of IL-19 amino acid sequences. Dots indicate identity to Monodelphis domestica sequence. Sequences used for alignment: Homo sapiens (NP_037503.2), Mus musculus (NP_001009940.1). [file 1745-7580-2-4-S9.doc]

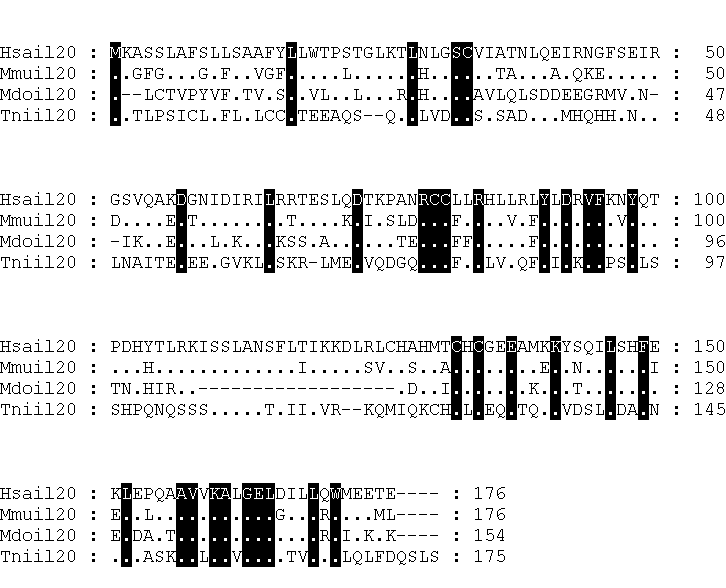

Supplement: Additional file 10 — Alignment of IL-20 amino acid sequences. Dots indicate identity to Monodelphis domestica sequence. Sequences used for alignment: Homo sapiens (NP_061194.2), Mus musculus (NP_067355.1), Tetraodon nigroviridis (AAP57416.1). [file 1745-7580-2-4-S10.doc]

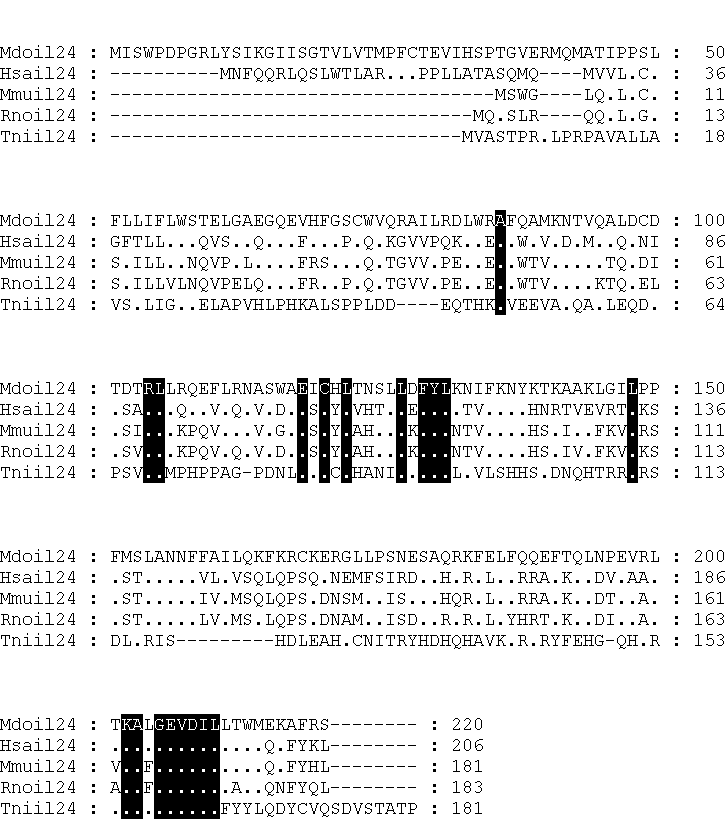

Supplement: Additional file 11 — Alignment of IL-24 amino acid sequences. Dots indicate identity to Monodelphis domestica sequence. Sequences used for alignment: Homo sapiens (NP_006841.1), Mus musculus (NP_444325.1), Rattus norvegicus (NP_579845.1), Tetraodon nigroviridis (AAP57418.1). [file 1745-7580-2-4-S11.doc]

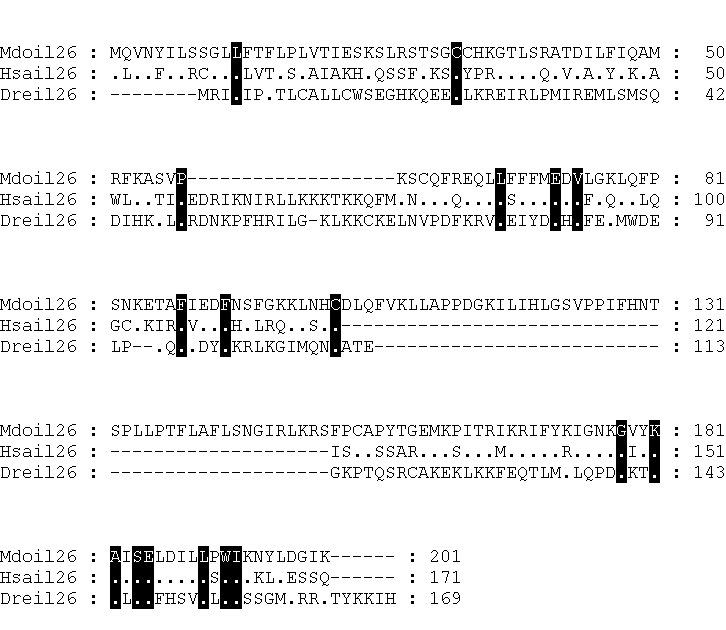

Supplement: Additional file 12 — Alignment of IL-26 amino acid sequences. Dots indicate identity to Monodelphis domestica sequence. Sequences used for alignment: Homo sapiens (NP_060872.1), Danio rerio (NP_001018635.1). [file 1745-7580-2-4-S12.doc]

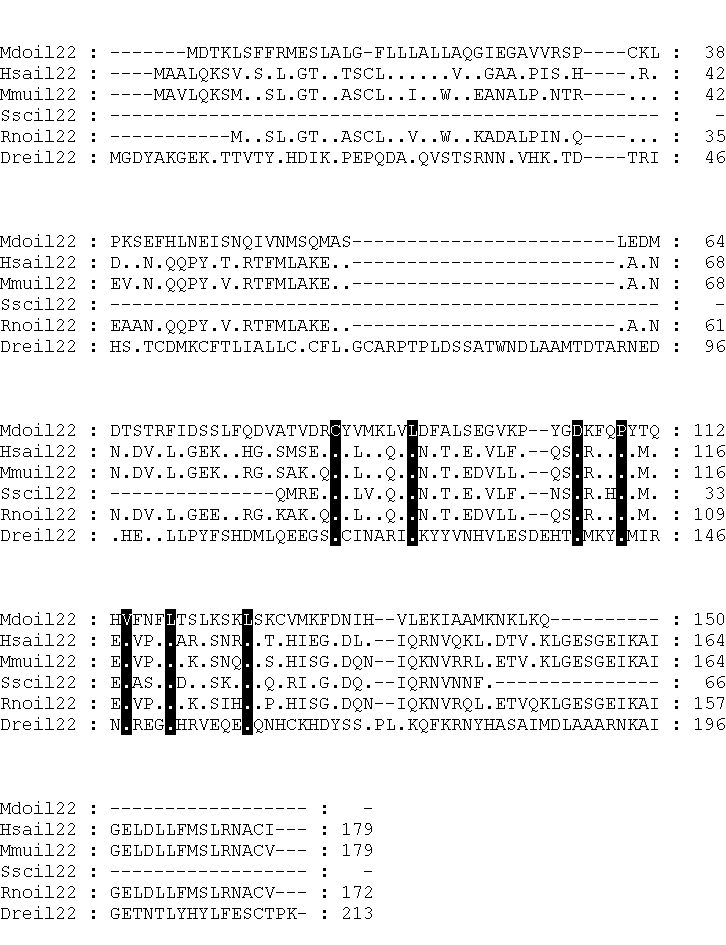

Supplement: Additional file 13 — Alignment of IL-22 amino acid sequences. Dots indicate identity to Monodelphis domestica sequence. Sequences used for alignment: Homo sapiens (NP_065386.1), Mus musculus (NP_058667.1), Sus scofa (AAX33671.1), Rattus norvegicus (ABF82262.1), Danio rerio (NP_001018628.1). [file 1745-7580-2-4-S13.doc]

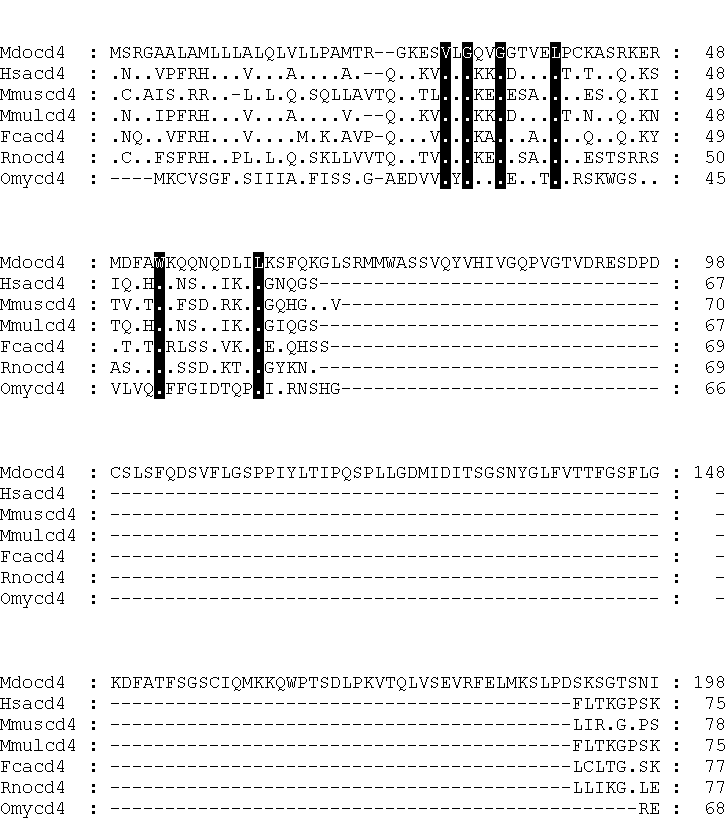


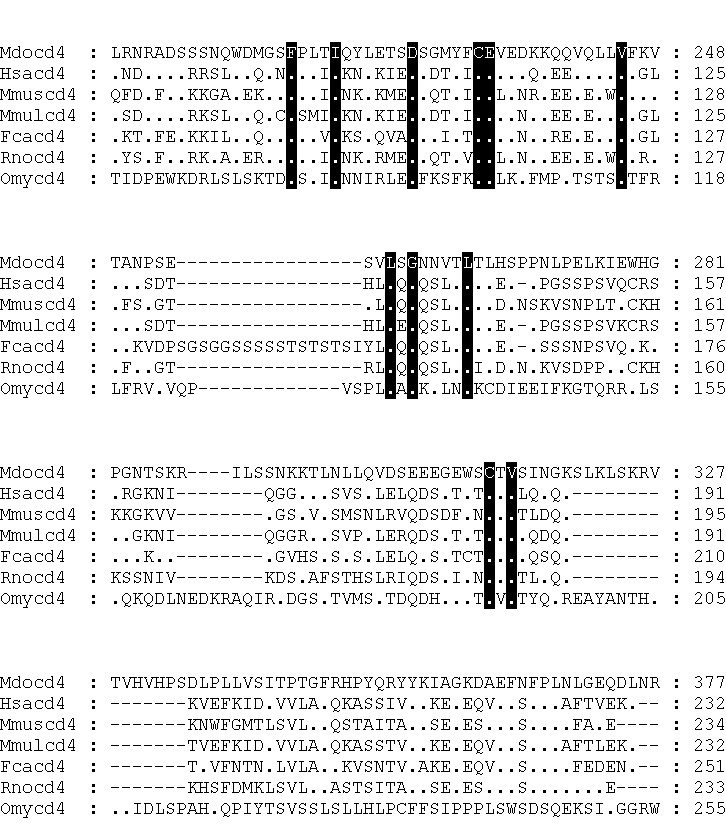


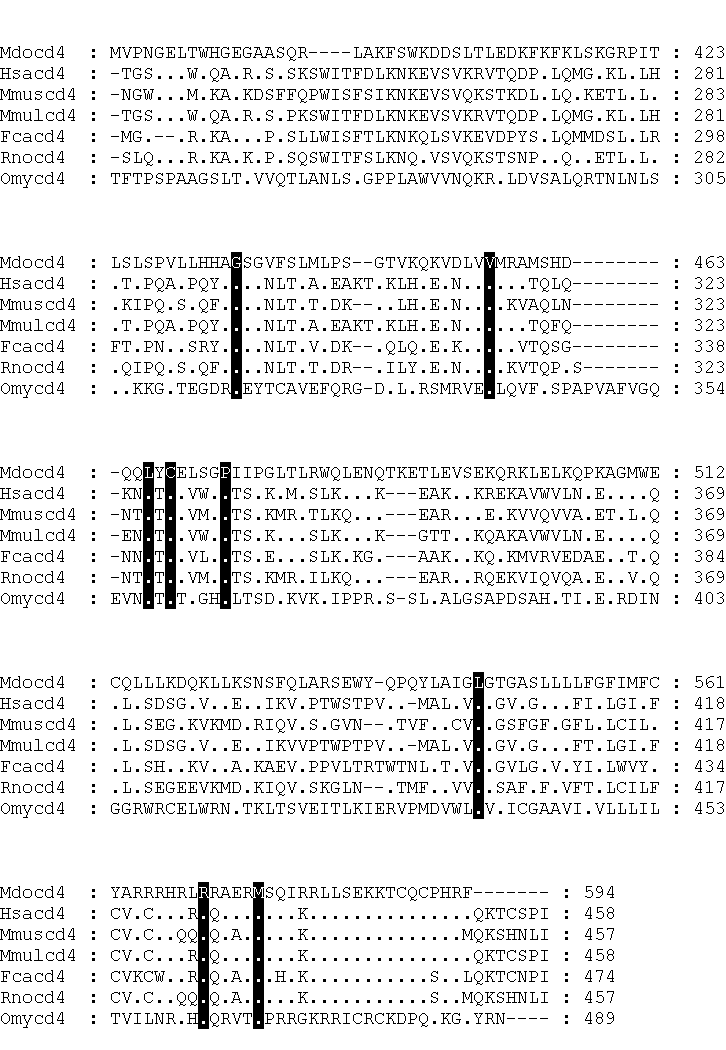

Supplement: Additional file 15 — Alignment of CD4 amino acid sequences. Dots indicate identity to Monodelphis domestica sequence. Sequences used for alignment: Homo sapiens (NP_000607.1), Mus musculus (NP_038516.1), Macaca mulatta (BAA09671.1) Felis cattus (NP_001009250.1), Rattus norvegicus (NP_036837.1) Oncorhynchus mykiss (AAY42068.1). [file 1745-7580-2-4-S15.doc]

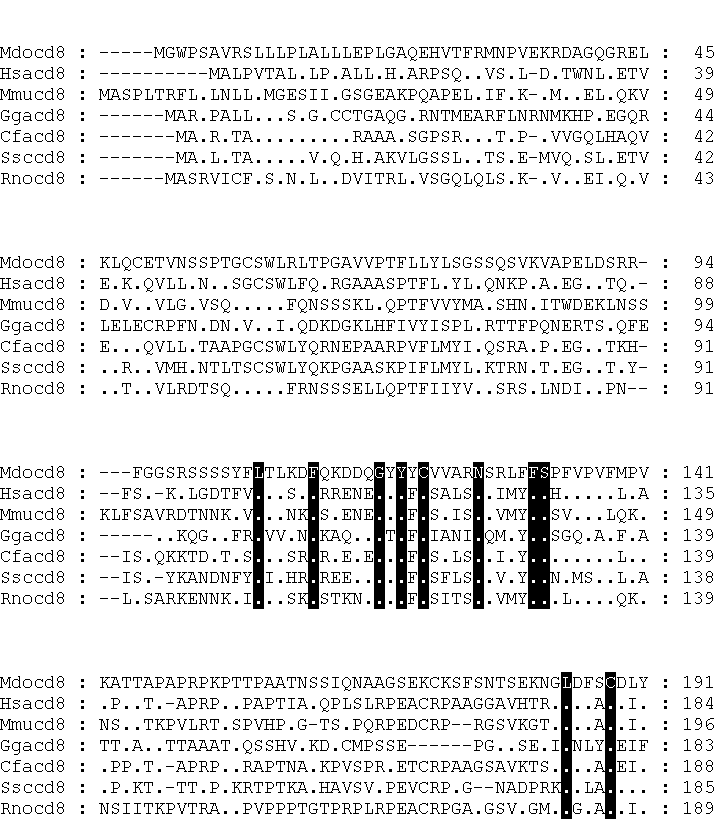


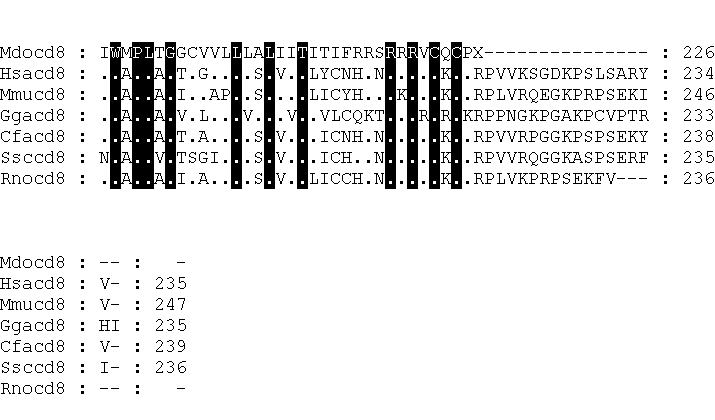

Supplement: Additional file 16 — Alignment of CD8 amino acid sequences. Dots indicate identity to Monodelphis domestica sequence. Sequences used for alignment: Homo sapiens (NP_001759.3), Mus musculus (Q60965), Gallus gallus (NP_990566.1), Canis familiaris (NP_001002935.1), Sus scofa (NP_001001907.1), Rattus norvegicus (AAH88126.1). [file 1745-7580-2-4-S16.doc]

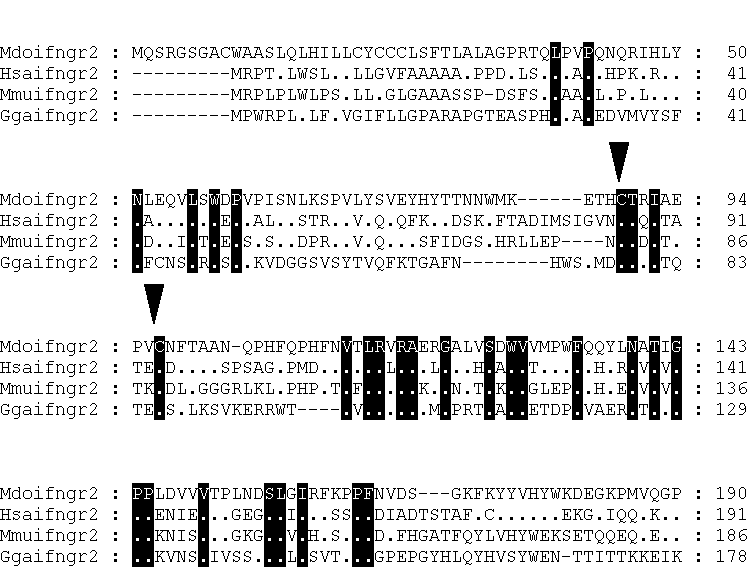


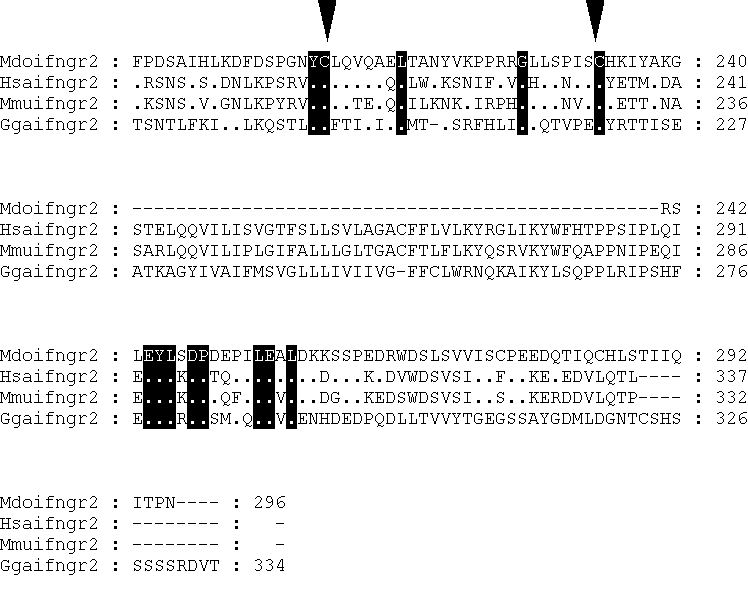

Supplement: Additional file 17 — Alignment of IFNGR-2 amino acid sequences. Cysteine residues conserved among all species are marked with an inverted triangle. Dots indicate identity to Monodelphis domestica sequence. Sequences used for alignment: Homo sapiens (NP_005525.2), Mus musculus (NP_032364.1), Gallus gallus (NP_001008676.1). [file 1745-7580-2-4-S17.doc]
