# Supplementary material for: In silico identification of opossum cytokine genes suggests the complexity of the marsupial immune system rivals that of eutherian mammals
Source: Immunome Res. 2006 Nov 10;2:4. doi: 10.1186/1745-7580-2-4 (PMC1660534; doi:10.1186/1745-7580-2-4)
Supplement: Supplementary file 2 [file 1745-7580-2-4-S14.doc]

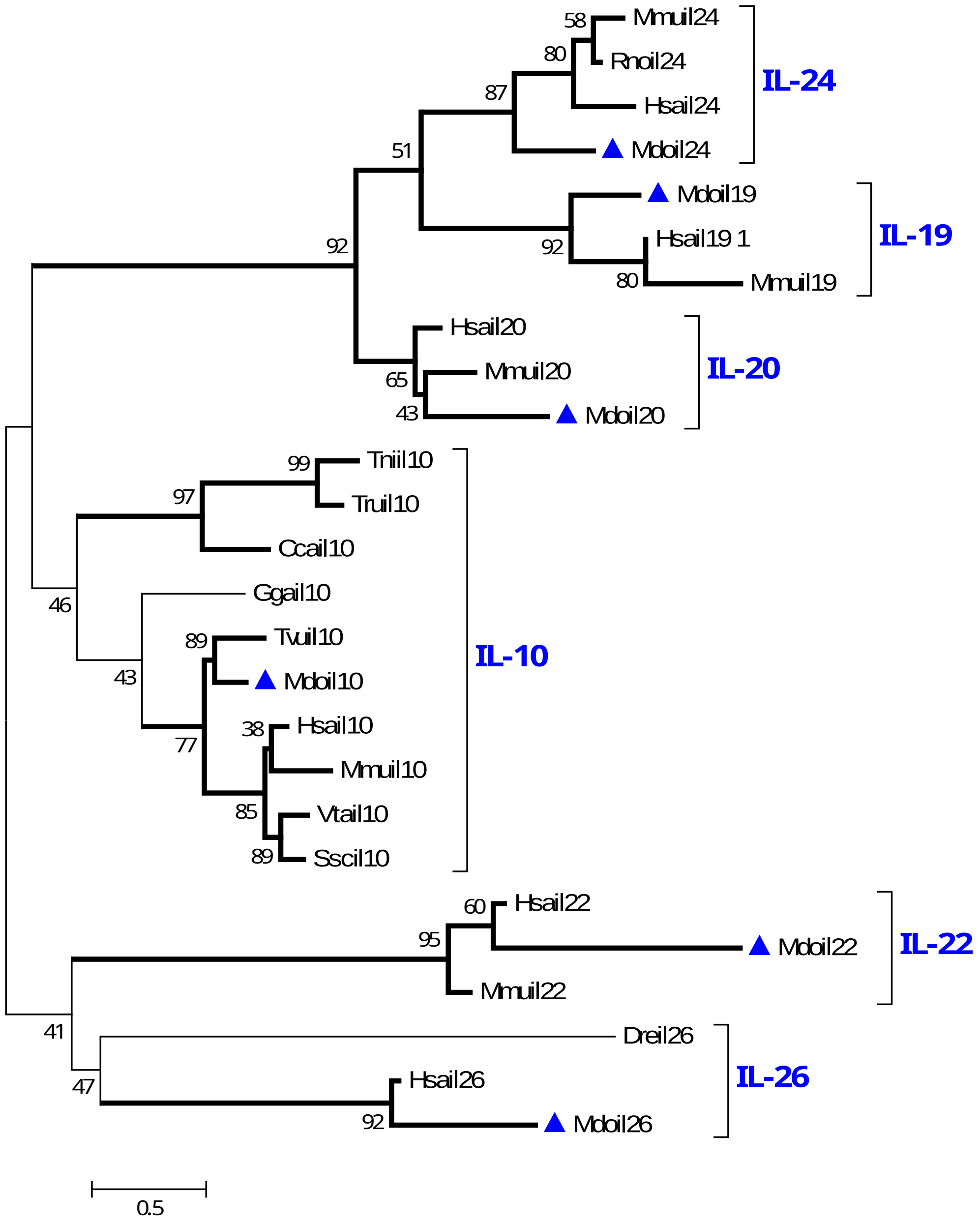

Mmuil24
Rnoil24
Hsail24
Mdoil24
IL-24
Mdoil19
Hsail19 1
Mmuil19
IL-19
Hsail20
Mmuil20
Mdoil20
IL-20
Tniil10
Truil10
Ccail10
Ggail10
Tvuil10
Mdoil10
Hsail10
Mmuil10
Vtail10
Sscil10
IL-10
Hsail22
Mdoil22
Mmuil22
IL-22
Dreil26
Hsail26
Mdoil26
IL-26
58
80
87
51
92
92
80
65
43
99
97
46
43
89
77
38
85
89
60
95
41
47
92
0.5
